# Circulating lnc-LOC as a novel noninvasive biomarker in the treatment surveillance of acute promyelocytic leukaemia

**DOI:** 10.1186/s12885-022-09621-1

**Published:** 2022-05-02

**Authors:** Guiran Wang, Guiling Yan, Kanru Sang, Huijie Yang, Ni Sun, Yuanyuan Bai, Feng Xu, Xiaoqun Zheng, Zhanguo Chen

**Affiliations:** 1 Department of Clinical Laboratory, The Second Affiliated Hospital and Yuying Children’s Hospital of Wenzhou Medical University, 109 Xueyuan Xi Road, Wenzhou, Zhejiang 325000 P.R. China; 2 The First School of Clinical Medicine, Wenzhou Medical University, Wenzhou, Zhejiang 325000 P.R. China; 3 Department of Clinical Laboratory, Fengxian Hospital Affiliated to Southern Medical University, Nanfeng Road 6600, Shanghai, 201499 P.R. China; 4 Department of Haematology, The Second Affiliated Hospital and Yuying Children’s Hospital of Wenzhou Medical University, Wenzhou, Zhejiang 325000 P.R. China; 5 School of Laboratory Medicine and Life Sciences, The Key Laboratory of Laboratory Medicine, Wenzhou Medical University, Ministry of Education of China, Wenzhou, Zhejiang 325035 P.R. China

**Keywords:** Lnc-LOC, Acute promyelocytic leukaemia, Minimal residual disease, Noninvasive biomarker, Surveillance

## Abstract

**Background:**

Acute promyelocytic leukaemia (APL) is a unique subtype of acute myeloid leukaemia (AML) characterized by haematopoietic failure caused by the accumulation of abnormal promyelocytic cells in bone marrow (BM). However, indispensable BM biopsy frequently afflicts patients in leukaemia surveillance, which increases the burden on patients and reduces compliance. This study aimed to explore whether the novel circulating long noncoding RNA LOC100506453 (lnc-LOC) could be a target in diagnosis, assess the treatment response and supervise the minimal residual disease (MRD) of APL, thereby blazing a trail in noninvasive lncRNA biomarkers of APL.

**Methods:**

Our study comprised 100 patients (40 with APL and 60 with non-APL AML) and 60 healthy donors. BM and peripheral blood (PB) sample collection was accomplished from APL patients at diagnosis and postinduction. Quantitative real-time PCR (qRT–PCR) was conducted to evaluate lnc-LOC expression. A receiver operating characteristic (ROC) analysis was implemented to analyse the value of lnc-LOC in the diagnosis of APL and treatment monitoring. For statistical analysis, the Mann–Whitney U test, a t test, and Spearman’s rank correlation test were utilized.

**Results:**

Our results showed that BM lnc-LOC expression was significantly different between APL and healthy donors and non-APL AML. lnc-LOC was drastically downregulated in APL patients’ BM after undergoing induction therapy. Lnc-LOC was upregulated in APL cell lines and downregulated after all-trans retinoic acid (ATRA)-induced myeloid differentiation, preliminarily verifying that lnc-LOC has the potential to be considered a treatment monitoring biomarker. PB lnc-LOC was positively correlated with BM lnc-LOC in APL patients, non-APL AML patients and healthy donors and decreased sharply after complete remission (CR). However, upregulated lnc-LOC was manifested in relapsed-refractory patients. A positive correlation was revealed between PB lnc-LOC and PML-RARα transcript levels in BM samples. Furthermore, we observed a positive correlation between PB lnc-LOC and BM lnc-LOC expression in APL patients, suggesting that lnc-LOC can be utilized as a noninvasive biomarker for MRD surveillance.

**Conclusions:**

Our study demonstrated that PB lnc-LOC might serve as a novel noninvasive biomarker in the treatment surveillance of APL, and it innovated the investigation and application of newly found lncRNAs in APL noninvasive biomarkers used in diagnosis and detection.

**Supplementary Information:**

The online version contains supplementary material available at 10.1186/s12885-022-09621-1.

## Background

Acute promyelocytic leukaemia (APL) is a unique subtype of acute leukaemia characterized by balanced chromosomal ectopic t (15;17) (q22; q12), leading to promyelocytic (PML) genes and retinol receptor alpha (RARα) gene fusion [1]. Although the combination of all-trans retinoic acid (ATRA) and chemotherapy has proven to be very effective in the de novo treatment of APL patients, relapse still occurs in approximately 10% of patients. Therefore, early diagnosis and prompt treatment are particularly important [2, 3]. Monitoring minimal residual disease (MRD) in APL patients has been established as a vital survival prognostic factor. MRD monitoring of bone marrow (BM) has been applied in routine clinical practice for all patients. However, BM aspirates are invasive, which burdens patients in leukaemia surveillance [4–6]. Therefore, more novel noninvasive biomarkers are urgently needed for prompt diagnosis and treatment monitoring during long-term therapy. Studies have shown that MRD assessment by qPCR in peripheral blood (PB) is an informative tool for disease surveillance in childhood acute myeloid leukaemia (AML) and provides the conditions for pre-emptive therapy, which suggests that PB has the potential to serve as a sample source [7]. Although BM is considered the gold standard sample source for diagnosis and MRD surveillance in APL, PB has advantages in terms of minimal invasion and long-term monitoring.

Previous works have shown that circulating tumour nucleic acids, circulating tumour cells and exosomes can be applied to liquid biopsy and are highly likely to become part of future clinical practice [8, 9]. MRD is a valuable marker for evaluating the treatment response, and MRD surveillance is recommended as part of the standard of care for AML patients [10]. Long noncoding RNAs (lncRNAs), defined as transcripts with lengths exceeding 200 nucleotides, have scarcely any capacity to translate into proteins but play a crucial role in numerous important biological phenomena with cellular and tissue specificity [11]. Deregulation of lncRNAs has been observed in various cancers, which suggests that lncRNAs may act as potential targets for therapy in cancer [11, 12]. In addition, many studies have highlighted that some lncRNAs steadily persist in human circulation [13], which enables the probable detection of disease diagnosis and prognosis biomarkers. Furthermore, lncRNAs SOX2OT and ANRIL are ideal prognostic biomarkers for non-small-cell lung cancer [14]. lncRNA GIHCG serves as a novel diagnostic and prognostic biomarker and therapeutic target for renal cell carcinoma [15]. Previous investigations have demonstrated that lncRNAs are crucial in myeloid differentiation and APL therapy [16, 17]. HOTAIRM1 regulates autophagy and the degradation of the PML-RARα oncoprotein in APL [18]. However, whether lncRNAs can be potential biomarkers in APL remains largely undefined.

The identification of dysregulated lncRNAs may provide new targets for the diagnosis and treatment of APL. Based on previous studies, we systematically integrated the gene expression profiles of APL and significantly coexpressed gene pairs by using GeneChip analysis. Then, we first selected one candidate differentially expressed long noncoding RNA, LOC100506453 (lnc-LOC), whose gene symbol is LOC100506453 and gene accession number is ENST00000424415, in BM samples from APL patients with ATRA-based targeted therapy [19]. However, further exploration of whether PB lnc-LOC represents the ideal biomarker to substitute the gold standard harvested from BM still needs to be conducted.

In this study, we investigated lnc-LOC expression in APL by establishing a sensitive and specific TaqMan probe-based quantitative real-time PCR (qRT–PCR) and then assessed its potential value. Our data showed that after the induction of ATRA, the expression of lnc-LOC was downregulated in both APL cell lines (NB_4_ cells and HL-60 cells) and clinical samples from patients. Moreover, therapeutic response and MRD surveillance can be accurately monitored through PB lnc-LOC detection, which is quite favourable for the noninvasive monitoring of APL. We aim to provide a novel noninvasive biomarker in the treatment surveillance of APL.

## Materials and methods

### Patient profiles

In this study, we enrolled 100 leukaemia patients, including 40 APL patients and 60 non-APL AML patients, from The Second Affiliated Hospital and Yuying Children’s Hospital of Wenzhou Medical University between April 2018 and October 2019 (Table 1). All leukaemia cases met the WHO 2016 acute leukaemia classification criteria (Table 2) [20]. For comparison, we also enrolled 60 sex- and age-matched healthy donors without haematopoietic malignancies as controls. Inclusion criteria: Newly diagnosed APL and non-APL AML patients did not receive any differentiation therapy. APL patients received induction therapy and follow-up therapy according to the NCCN guidelines, and molecular complete remission (CR) was defined according to standard criteria [21]. PB and BM samples were also collected from all APL patients at diagnosis and postinduction, non-APL AML patients, healthy donors and 5 relapsed APL patients during follow-up. This study obtained informed consent from all patients and was approved by the Ethics Committee of The Second Affiliated Hospital and Yuying Children’s Hospital of Wenzhou Medical University.

**Table 1:** Characteristics of 100 patients with AML

Variable at diagnosis	APL patients (***n*** = 40)	Non-APL patients (***n*** = 60)
	N (%)	N (%)
**Gender**		
Female	25 (62.5)	30 (50)
Male	15 (37.5)	30 (50)
**Age**		
**<** 50	30 (75.0)	35 (58.3)
≥ 50	10 (25.0)	25 (41.7)
**Leukocyte counts, ×10**^**9**^**/L**		
**<** 10	35 (87.5)	45 (75.0)
≥ 10	5 (12.5)	15 (25.0)
**Risk group**		
Low/intermediate	35 (87.5)	**–**
High	5 (12.5)	**–**
**Treatment**		
ATO **+** ATRA	38 (95.0)	**–**
Cytarabine + ATRA	2 (5.0)	**–**

Abbreviations: *AML* acute myeloid leukaemia, *APL* acute promyelocytic leukaemia, *ATO* Arsenic Trioxide, *ATRA* all-trans retinoic acid

**Table 2:** WHO classification of 100 patients with AML

WHO classification	N (%)
APL with PML-RARα	40 (40.0)
AML with t(8;21)(q22;q22); RUNX1-RUNX1T1	10 (10.0)
AML with inv.(16)(p13.1q22) or t(16;16)(p13.1;q22); CBFB-MYH11	7 (7.0)
AML with t(9;11)(p21.3;q23.3); MLLT3-KMT2A	12 (12.0)
AML with minimal differentiation	6 (6.0)
Acute myelomonocytic leukaemia	25 (25.0)

Abbreviations: *AML* acute myeloid leukaemia, *APL* acute promyelocytic leukaemia

### Cell line and cell culture

The human leukaemia cell lines NB4, HL-60, THP-1, U937, K562, Kasumi-6 and HEL were purchased from the Shanghai Institution of Haematology and cultured in RPMI 1640 (Gibco BRL, USA) containing 10% foetal bovine serum (Gibco BRL, USA) at 37 °C in a humidified 5% CO_2_ incubator. Both APL cells (NB4) and non-APL cells were used for the ATRA treatment experiment. All cells were treated with a final concentration of 2 μM ATRA (Sigma–Aldrich, USA) or negative control dimethyl sulfoxide (DMSO) (Sigma–Aldrich, USA) and plated at a density of 2 × 10^5^ cells/well in 6-well plates for 24, 48 or 72 h, respectively. Lnc-LOC and PML-RARα expression in human leukaemia cell lines or ATRA-treated APL cells was determined by qRT–PCR.

### Sample collection and RNA extraction

PB samples and BM samples were collected from patients and healthy donors using EDTA-K_2_ anticoagulant blood vessels. Approximately 2 ml BM samples and PB samples were isolated using Density Reagent (QuantoBio, China) according to the manufacturer’s instructions. Total RNA was extracted from BM/PB samples or cell samples with TRIzol reagent (Invitrogen, USA) according to the manufacturer’s protocol, and only those that showed a ratio of A260/A280 between 1.8 and 2.0 were used in reverse transcription for cDNA [22]. All cells were temporarily stored at − 80 °C until further analysis. cDNA was synthesized with a RevertAid First Strand cDNA Synthesis Kit (Thermo Scientific, USA).

### Quantitative real-time polymerase chain reaction

qRT–PCR was performed with TaqMan Universal PCR Master Mix II, no UNG (Applied Biosystems, USA), and GAPDH was used as a reference for lncRNAs. After the extensive test, the final 20 μL optimized amplification system of lnc-LOC or GAPDH was as follows: 10 μL TaqMan Universal PCR Master Mix II, NO UNG (2×), 0.6 μL upstream primer (10 μM), 0.6 μL downstream primer (10 μM), 0.4 μL MGB probe (10 μM), 6.4 μL enzyme-free water, and 2 μL cDNA. Forward (F) and reverse (R) primers were synthesized by Shanghai Yiyue Biological Technology Company (Shanghai, China) as follows: lnc-LOC, lnc-LOC100506453-F GAGACCCTGGAAATAAAC, and lnc-LOC100506453-R CGATGGAATCAGTTAGAC; and GAPDH, hGAPDH-F TGCACCACCAACTGCTTAGC, and hGAPDH-R TCTTCTGGGTGGCAGTGATG. The probe sequences were as follows: lnc-LOC100506453-MGB FAM-TGGCTTCAGCGTCACCTAGT-MGB and GAPDH-MGB FAM- ACTGTGGTCATGAGTC-MGB. The PCR program was run as follows: denaturation at 95 °C for 10 min, 45 cycles of extension at 95 °C for 5 s, and 59 °C for 1 min. qRT–PCR was repeated three times. The expression level relative to the control samples was calculated by the 2^−ΔΔCt^ method [4].

### MRD analysis

MRD analysis was based on 40 BM samples collected from APL patients before and after induction therapy. qRT–PCR was used to detect PML-RARα fusion gene transcripts from all BM samples on an ABI7500 System with a TaqMan probe as previously described [23]. According to the Europe against Cancer (ECA) program, the MRD value was presented as the absolute [4, 24]. ABL was used as a control gene (CG) to normalize PML-RARα fusion transcripts to generate a normalized copy number (NCN) [25].

### Statistical analysis

SPSS 21.0 was used for statistical analysis, and graph plotting was performed using GraphPad Prism 8.01 (GraphPad Software, USA). The nonparametric Mann–Whitney U test and a t test were performed to assess the differences in lnc-LOC expression between experimental samples and control samples. The receiver operating characteristic (ROC) curve was used to evaluate the value of lnc-LOC for APL diagnosis. Spearman’s rank correlation test and rank-sum test were performed for the correlation analysis. *P* < 0.05 was considered statistically significant.

## Results

### Genetic characteristic of lnc-LOC

Based on previous studies, we explored some important lncRNAs in APL patients with ATRA-based targeted therapy through a combined computational and experimental approach [19]. Finally, we selected lnc-LOC as an essential lncRNA for further study. The gene accession number of lnc-LOC was ENST0000042441. lnc-LOC with 2194 base pairs (bp) was located on chromosome X: 3,809,479-3,820,041 reverse strands. The details of lnc-LOC are listed in Supplemental Table 1.

### BM lnc-LOC as a potential biomarker for APL diagnosis

**Fig. 1:**
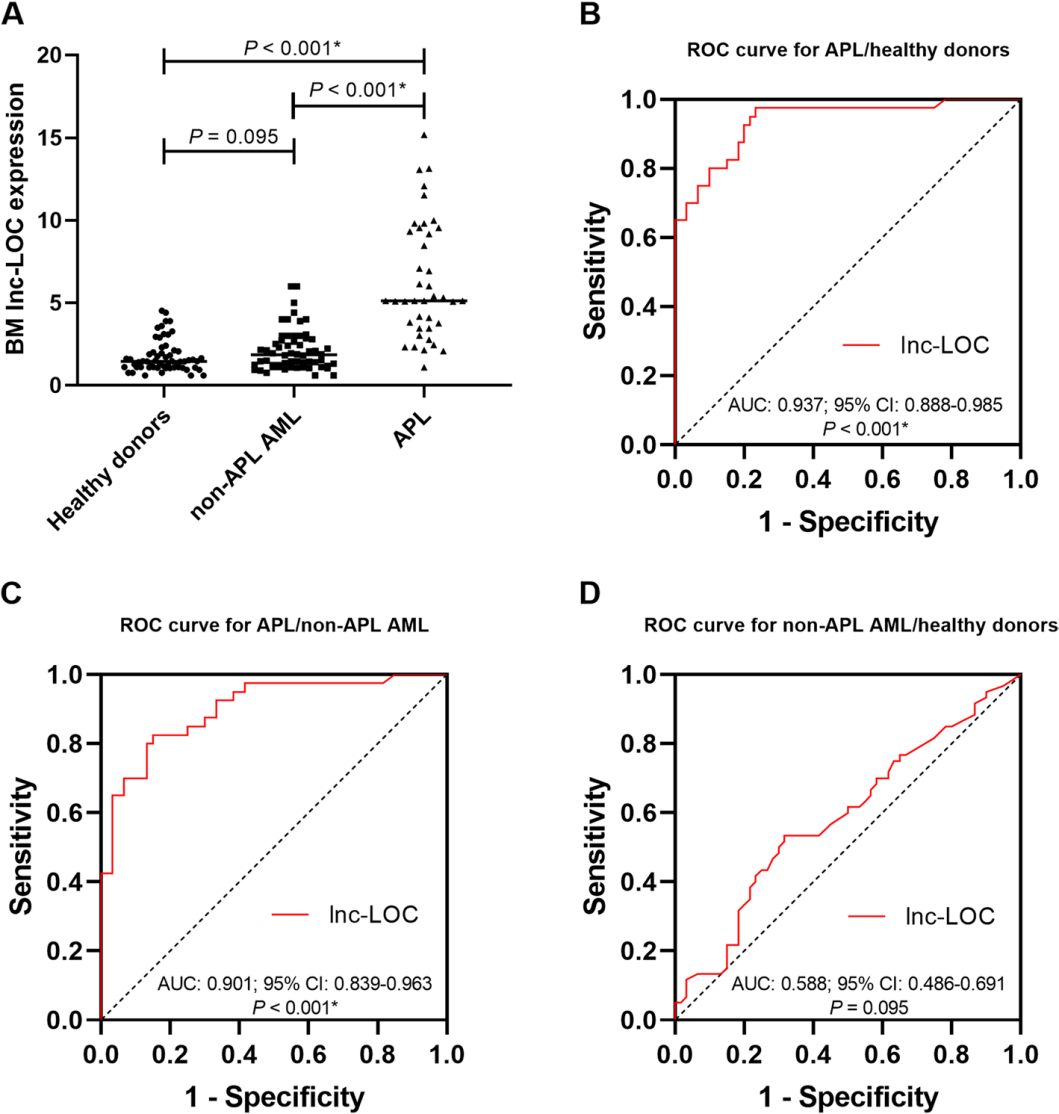
BM lnc-LOC expression and diagnostic value in APL patients. **a** Comparison of BM lnc-LOC expression in different groups, including APL, non-APL AML and healthy donors. **b** ROC curve analysis of BM lnc-LOC expression for the discrimination of APL from healthy donors. **c** ROC curve analysis of lnc-LOC expression for the discrimination of APL from non-APL AML. **d** ROC curve analysis of lnc-LOC expression for the discrimination of non-APL AML from healthy donors. * Statistically significant

To confirm whether BM lnc-LOC is a potential biomarker for APL diagnosis, we used qRT–PCR to detect BM lnc-LOC expression in APL, non-APL AML and healthy donors. Representative amplification plots of APL, non-APL AML, and healthy donors by qRT–PCR are shown in Supplemental Fig. 1. The results suggested that lnc-LOC expression was higher in BM samples from APL patients than in BM samples from non-APL AML patients or healthy donors (*P* < 0.001; Fig. 1A). To investigate whether BM lnc-LOC expression was qualified for APL diagnosis, we used ROC curve analysis to reveal the diagnostic accuracy of lnc-LOC. When the optimal cut-off values were 2.070, 3.010 and 1.790, the areas under the ROC curves (AUCs) were 0.937 (95% CI = 0.888–0.985, *P* < 0.001; sensitivity = 0.975; specificity = 0.767) for distinguishing APL from healthy donors, 0.901 (95% CI = 0.839–0.963, *P* < 0.001; sensitivity = 0.825; specificity = 0.850) for distinguishing APL from non-APL AML, and 0.588 (95% CI = 0.486–0.691, *P* = 0.095; sensitivity = 0.533; specificity = 0.683) for distinguishing non-APL AML from healthy donors (Fig. 1B-D). Taken together, BM lnc-LOC expression was significantly different between APL and healthy donors and non-APL AML, which indicated that BM lnc-LOC might be a potential biomarker for APL diagnosis.

### The specific expression of lnc-LOC in different leukaemia cell lineages and ATRA-treated APL cells

**Fig. 2:**
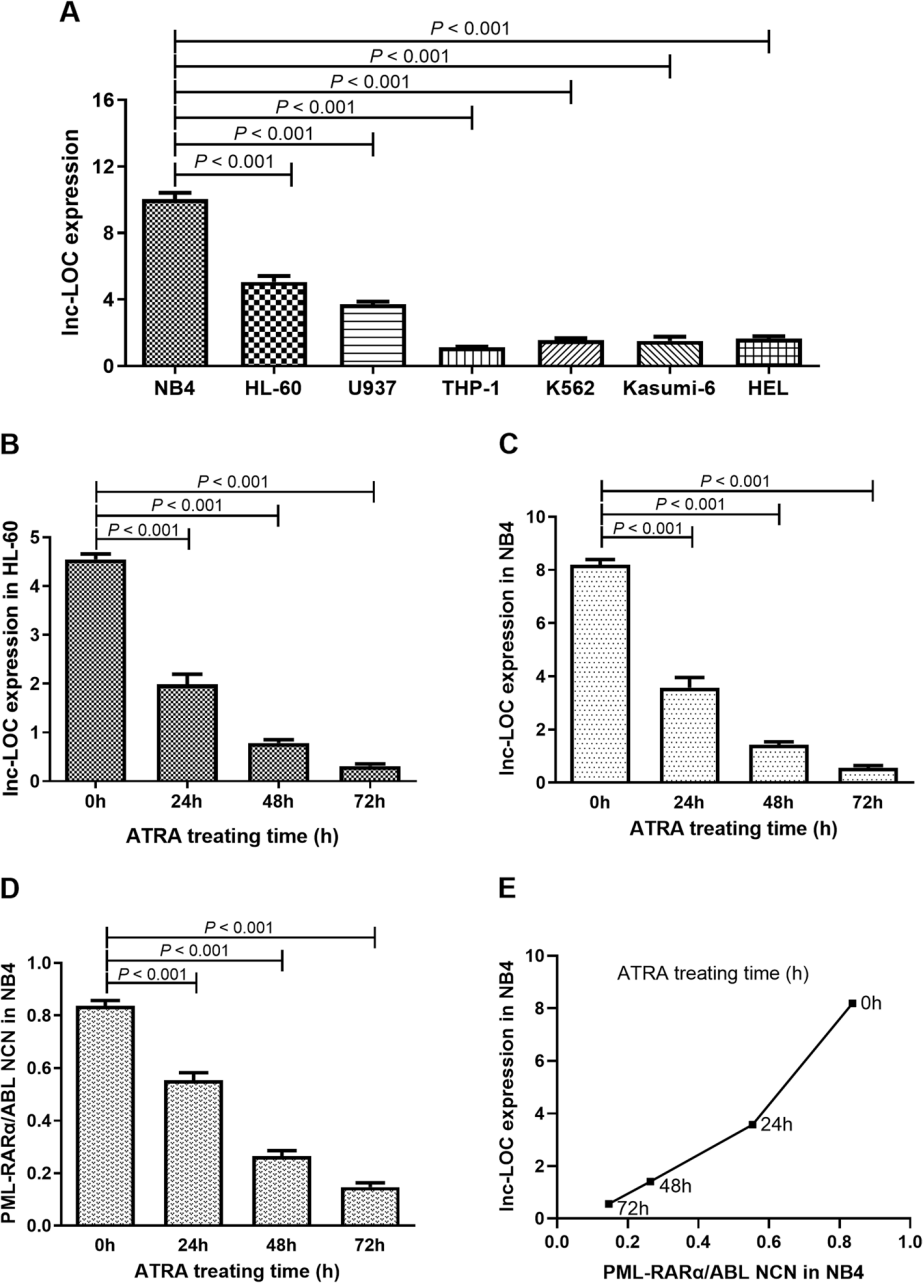
Differential expression of lnc-LOC in myeloid lineage and ATRA-treated APL cells. **a** lnc-LOC expression in myeloid lineage cells, including NB4, HL-60, U937, THP-1, K562, Kasumi-6, and HEL cells. **b** lnc-LOC expression in HL-60 cells treated with ATRA as indicated. **c** lnc-LOC expression in NB4 cells treated with ATRA as indicated. **d** PML-RARα transcript expression in NB4 cells treated with ATRA as indicated. **e** lnc-LOC expression in NB4 cells was positively correlated with PML-RARα transcript expression after ATRA treatment in NB4 cells. * Statistically significant

To clarify the specific expression of lnc-LOC in the development of haematopoietic cells, we assessed lnc-LOC expression in different leukaemia cell lineages by qRT–PCR. The results showed that lnc-LOC expression was significantly higher in NB4 cells than in non-APL cells (HL-60, U937, THP-1, K562, Kasumi-6 and HEL) (*P* < 0.001; Fig. 2A). Next, we selected NB4 and HL-60 cells as models of BM differentiation. Lnc-LOC was downregulated in ATRA-treated HL-60 (*P* < 0.001; Fig. 2B) and NB4 cells (*P* < 0.001; Fig. 2C). Besides, the expression of PML-RARα in NB4 after ATRA treatment was downregulated (*P* < 0.001; Fig. 2D), and the expression of lnc-LOC was positively correlated with PML-RARα in NB4 after ATRA treatment (Fig. 2E). However, it was not observed in other non-APL cell lines (*P* > 0.05; Supplemental Fig. 2). In summary, lnc-LOC was specifically upregulated in APL cell lines and downregulated in ATRA-induced myeloid differentiation, suggesting that lnc-LOC could be used to measure the response to ATRA-based APL therapy.

### Correlation of lnc-LOC expression between PB samples and BM samples from APL patients

**Fig. 3:**
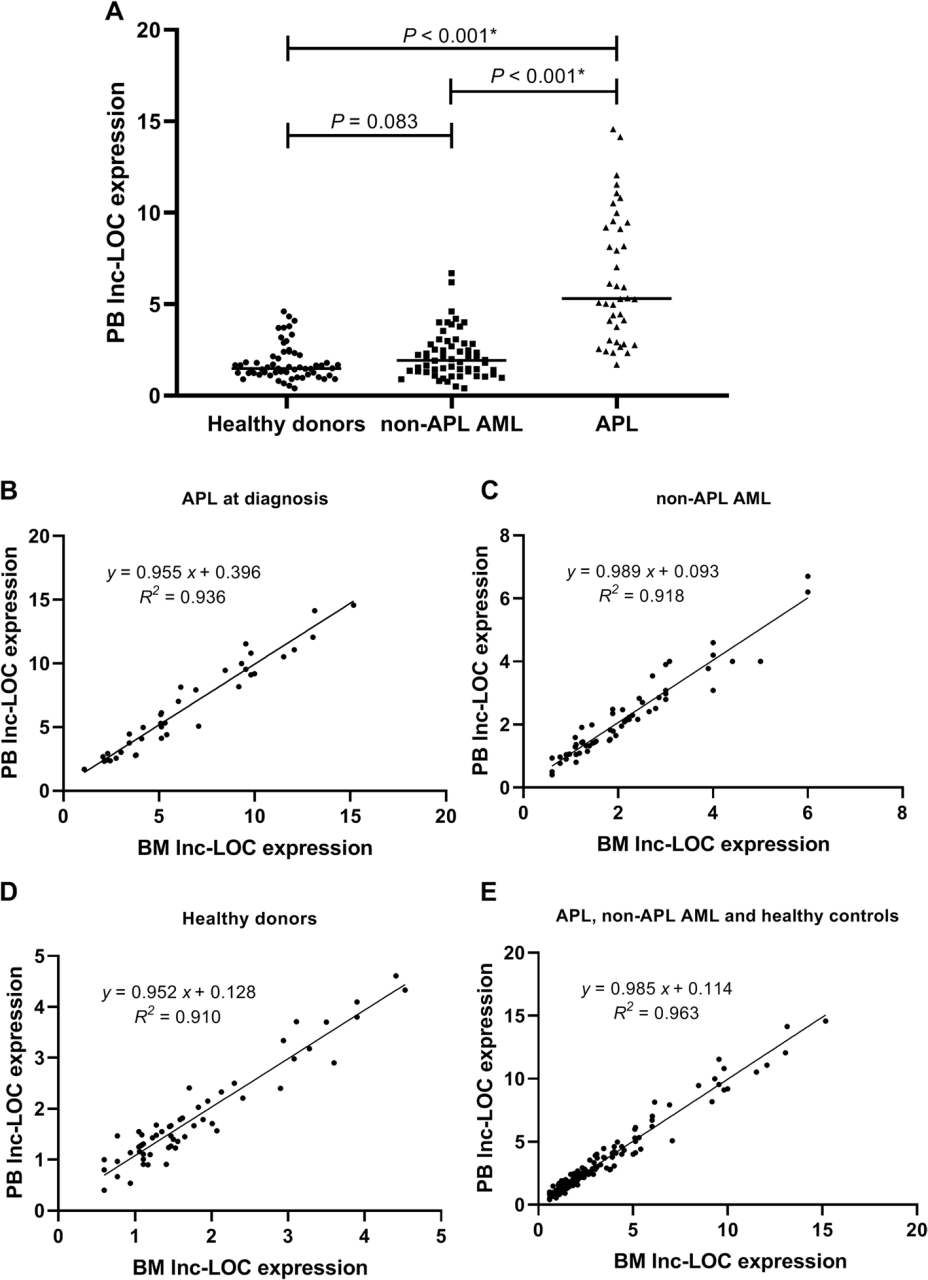
Correlation between lnc-LOC expression in PB samples and that in BM samples from APL patients. **a** Comparison of PB lnc-LOC expression in APL, non-APL AML and healthy donors. **b** PB lnc-LOC expression was positively correlated with BM lnc-LOC expression in APL at diagnosis (*R*^2^ = 0.936, *P* < 0.001*). **c** PB lnc-LOC expression was positively correlated with BM lnc-LOC expression in non-APL AML (*R*^2^ = 0.918, *P* < 0.001*). **d** PB lnc-LOC expression was positively correlated with BM lnc-LOC expression in healthy donors (*R*^2^ = 0.910, *P* < 0.001*). **e** A significant positive correlation was observed between all PB samples and BM samples in lnc-LOC expression (*R*^2^ = 0.963, *P* < 0.001*). * Statistically significant

To confirm whether PB samples can replace BM samples for lnc-LOC detection, we first evaluated PB lnc-LOC expression in different groups by qRT–PCR. As shown in Fig. 3A, PB lnc-LOC expression in the APL patient group was higher than that in either the non-APL AML patient group (*P* < 0.001) or the healthy donor group (*P* < 0.001). Then, we analysed the correlation between PB lnc-LOC and BM lnc-LOC expression in APL at diagnosis. The results showed a positive correlation between PB lnc-LOC and BM lnc-LOC expression in APL at diagnosis (*R*^2^ = 0.936, *P* < 0.001; Fig. 3B). We continued to study whether we could find similar results in non-APL AML patients and healthy donors. The results showed a good correlation between PB lnc-LOC and BM lnc-LOC expression in both non-APL AML (*R*^2^ = 0.918, *P* < 0.001; Fig. 3C) and healthy donors (*R*^2^ = 0.910, *P* < 0.001; Fig. 3D). In addition, we compared all PB samples with BM samples in lnc-LOC expression, and we obtained a similar conclusion (*R*^2^ = 0.963, *P* < 0.001; Fig. 3E). In summary, there was a good correlation between lnc-LOC expression in PB samples and in BM samples, suggesting that PB samples could be used instead of BM samples for lnc-LOC detection in APL patients.

### Utilization of PB lnc-LOCs in monitoring the treatment response of APL patients

**Fig. 4:**
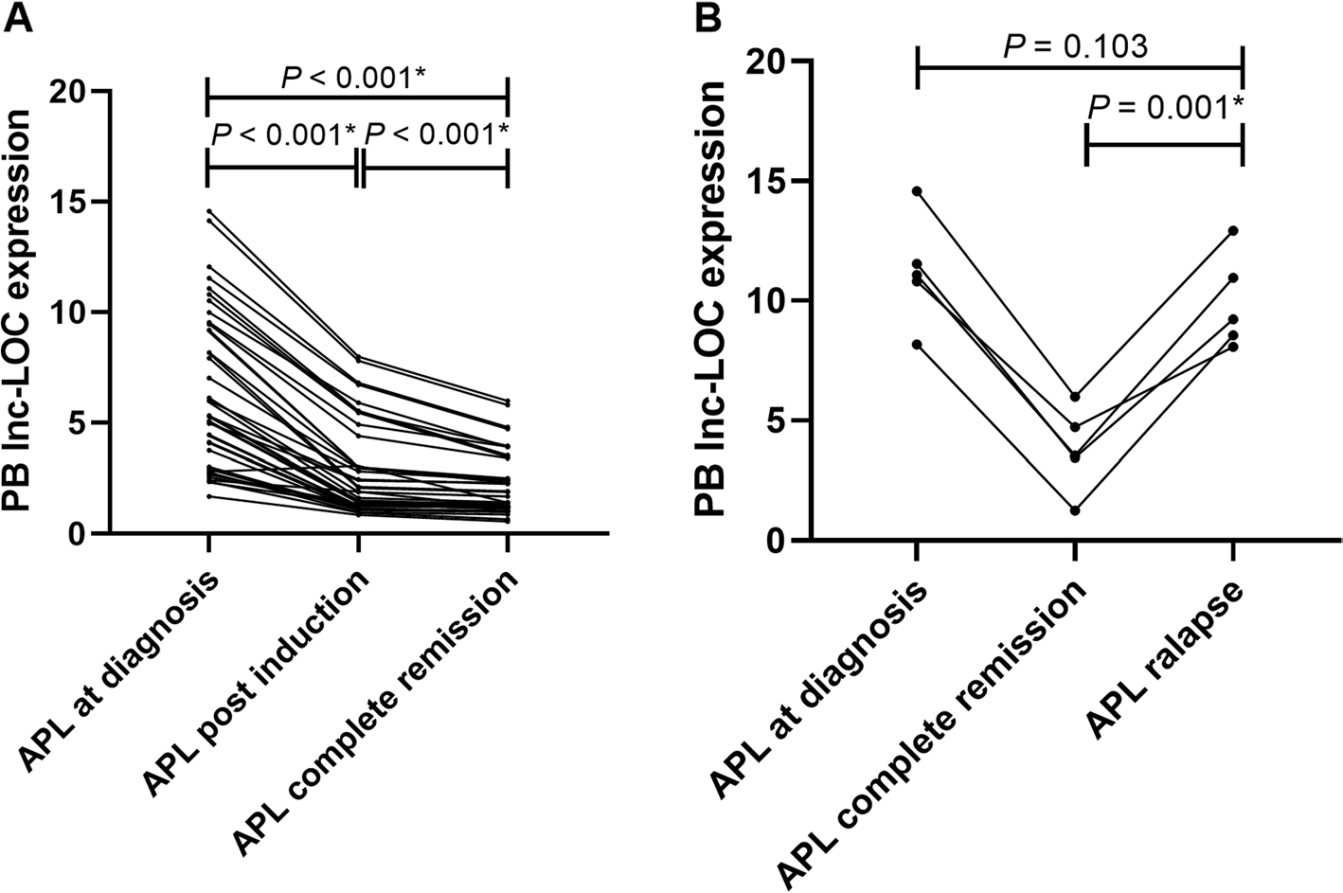
Utilization of PB lnc-LOC in monitoring the treatment response of APL patients. **a** PB lnc-LOC expression in 40 APL patients at new diagnosis, postinduction and CR. **b** PB lnc-LOC expression in 5 representative relapsed APL patients at different periods (at diagnosis, CR and relapse). * Statistically significant

To explore whether PB lnc-LOC can be applied for monitoring APL treatment response, we collected 40 pairs of PB samples from APL patients at initial diagnosis, after induction therapy and at the three-year clinical follow-up. The statistical results showed that after induction therapy, lnc-LOC expression decreased compared with newly diagnosed APL patients (*P* < 0.001; Fig. 4A). Moreover, lnc-LOC expression was continually reduced even after achieving remission (*P* < 0.001; Fig. 4A). Furthermore, lnc-LOC expression remained low every 3 months after remission (data not shown). However, when patients relapsed, lnc-LOC was upregulated (*P* = 0.001; Fig. 4B) and similar to the initial diagnosis (*P* = 0.103; Fig. 4B). Taken together, these results provide important insights into PB lnc-LOC, which could be a potential biomarker for treatment surveillance. Furthermore, these findings indicated that PB lnc-LOC downregulation after targeted therapy could reflect the response to treatment in APL.

### PB lnc-LOC as a noninvasive MRD surveillance marker for APL patients

**Fig. 5:**
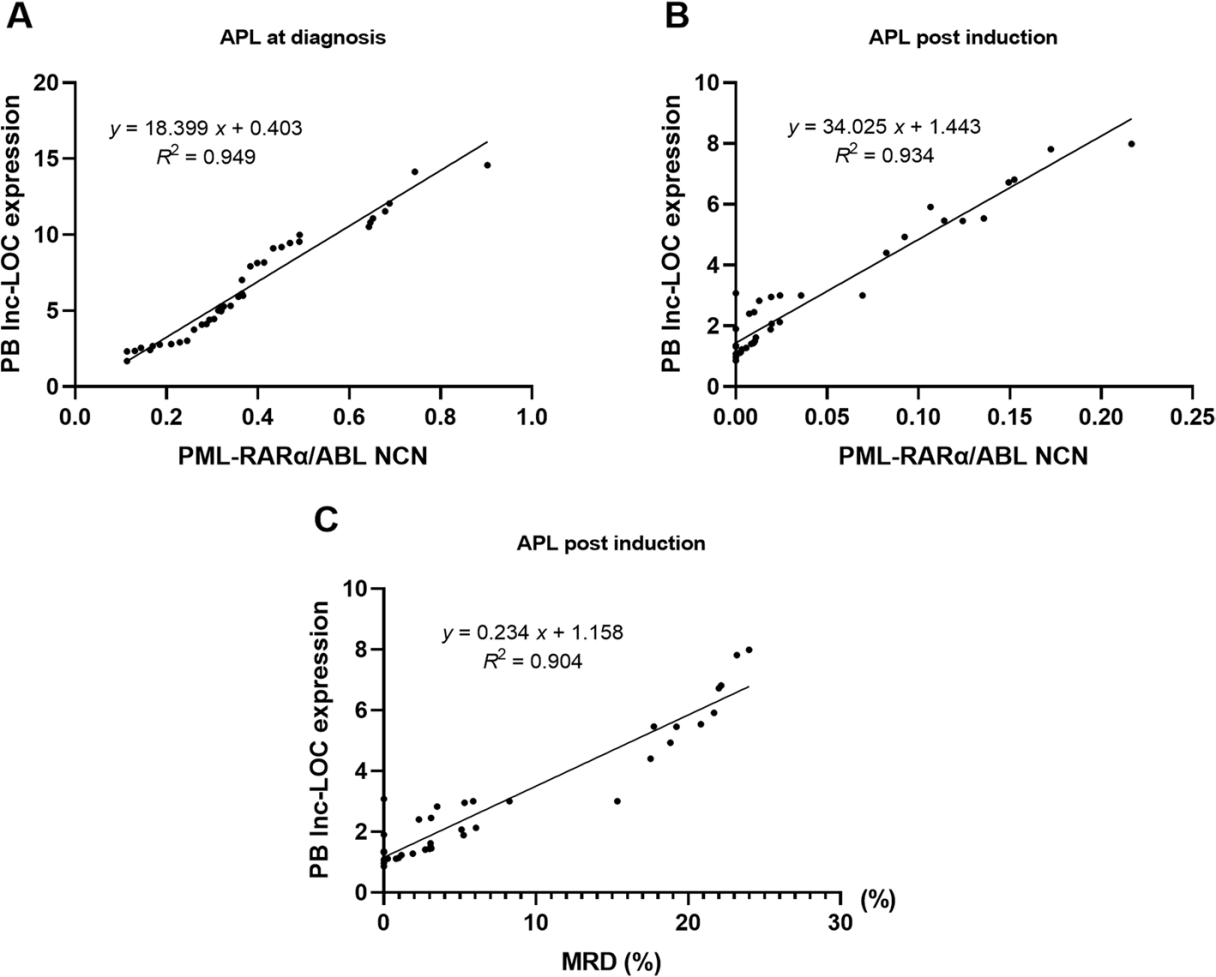
PB lnc-LOC as a potential MRD monitoring marker for APL patients. **a** PB lnc-LOC levels were positively correlated with PML-RARα transcript expression in APL patients at diagnosis (*R*^2^ = 0.949, *P* < 0.001*). **b** PB lnc-LOC levels were positively correlated with PML-RARα transcript expression in APL patients postinduction (*R*^2^ = 0.934, *P* < 0.001*). **c** PB lnc-LOC levels were consistently correlated with MRD values in APL patients postinduction (*R*^2^ = 0.904, *P* < 0.001*). * Statistically significant

To evaluate whether PB lnc-LOC can be used for MRD surveillance, we analysed the correlation between BM PML-RARα/ABL NCN and PB lnc-LOC before and after APL-induced differentiation. The results showed that lnc-LOC expression at diagnosis was positively correlated with PML-RARα/ABL NCN (*R*^2^ = 0.949, *P* < 0.001; Fig. 5A). We continued to study whether we could find similar results in APL-induced differentiation samples. The results showed a good correlation between BM PML-RARα/ABL NCN and PB lnc-LOC (*R*^2^ = 0.934, *P* < 0.001; Fig. 5B). In addition, lnc-LOC expression after induction was also positively correlated with the MRD value (*R*^2^ = 0.904, *P* < 0.001; Fig. 5C). This result suggested that PB lnc-LOC could reflect the MRD status of APL patients and can be utilized as a noninvasive MRD surveillance marker for APL patients.

## Discussion

In this study, we innovatively proposed lnc-LOC in PB samples, a newly found lncRNA, as a probable noninvasive biomarker for APL diagnosis and treatment surveillance. Our previous study found that lnc-LOC (primary ID ENST00000424415) from BM samples was a dysregulated lncRNA associated with ATRA-induced APL differentiation [19]. To investigate whether BM lnc-LOC might be a potential biomarker for APL diagnosis, we demonstrated higher expression of BM lnc-LOC in APL patients than in the non-APL group and the control group. Further experiments with ATRA-treated APL cells showed that lnc-LOC may be used to measure the response to ATRA-based APL therapy. Moreover, the correlation analysis of lnc-LOC expression in BM and PB samples indicated that PB samples could replace BM samples for lnc-LOC detection in APL patients. Based on the evidence mentioned above, we conducted subsequent investigations and found that the expression of lnc-LOC in PB significantly decreased in APL patients postinduction and post-CR, suggesting that downregulation of lnc-LOC in PB after targeted therapy could reflect the response of APL patients to treatment. Furthermore, in APL patients treated with ATRA-based targeted therapy, PB lnc-LOC was positively correlated with PML-RARα transcripts or MRD values in BM samples, indicating that PB lnc-LOC could reflect the MRD status of APL patients and may be used as a noninvasive treatment surveillance marker for APL.

Currently, technological advances and analysis of biomarkers provide new methods for haematological diseases, including APL. The common identification of APL-specific genetic lesions can be made by conventional karyotyping, fluorescence in situ hybridization (FISH), or comparable nucleic acid-based techniques [26]. However, research on noninvasive markers of APL is less common. Liquid biopsy has become the preferred choice for disease because of its noninvasiveness, low cost and high stability [27, 28]. However, there is still a research gap in applying circulating lncRNA biomarkers to APL diagnosis and surveillance.

lncRNAs have been demonstrated to have diverse functions in multiple biological processes and play important roles in cell differentiation and development, and it may implicate several cancers [29]. In colorectal cancer patients, upregulated PVT1 was positively correlated with cell proliferation, invasion, tumour stages, and lymph node metastasis [30]. lncRNA HOTAIR was found to be an independent prognostic biomarker of overall survival for transitional cell carcinoma (TCC) patients [31]. As a novel lncRNA, we discovered lnc-LOC through gene chip and bioinformatics analysis, and we first established and optimized the qRT–PCR method to detect lnc-LOC expression [32, 33]. Compared with healthy donors and non-APL AML patients, the expression of BM lnc-LOC was upregulated. In addition, ROC curve analysis suggests that lnc-LOC can effectively distinguish APL and non-APL AML, indicating that lnc-LOC might be an appropriate differentiated detection indicator for APL.

In a previous study, early detection of APL molecular relapse using BM was the preferred approach [34]. Nevertheless, MRD monitoring through PB remains a reasonable, more convenient and more comfortable choice for APL patients [35]. Therefore, we studied the potential connection between lnc-LOC and PML-RARα. Studies have shown that a positive correlation between lnc-LOC and PML-RARα transcription levels can be observed in both BM and PB samples. Furthermore, BM lnc-LOC expression and PB lnc-LOC expression in APL patients before and after receiving treatment were positively correlated. These results all indicated that PB lnc-LOC may be a new type of therapeutic biomarker for APL, especially as a noninvasive monitor for APL treatment.

In our previous study, we proved that miR-638 may be an ideal novel target for APL diagnosis and long-term surveillance [36]. However, the single-miRNA molecule limitation in both sensitivity and specificity [37], the high extraction difficulty of miRNAs and the stringent amplification requirements for stem–loop RT–PCR prompted us to search for more appropriate noninvasive biomarkers in APL. Compared with miR-638, lnc-LOC contains more superior features. High sensitivity and specification are the fundamental demands and the most important evaluation criteria for circulating lncRNAs as diagnostic or prognostic biomarkers for clinical application. According to our results, the sensitivity and specificity were 0.975 and 0.767, respectively, with an AUC of 0.937 (95% CI = 0.888–0.985) from the ROC analysis, suggesting that lnc-LOC could serve as a biomarker to differentiate APL from healthy donors.

PB represents an attractive specimen source for MRD surveillance, allowing for frequent sampling attributed to easier sample collection. There are still scarce studies showing that PB lncRNAs may be detected as potential noninvasive biomarkers for leukaemia. In addition, PB lnc-LOC expression showed a significantly positive correlation with BM lnc-LOC expression, indicating lnc-LOC as a valid biomarker in noninvasive tracking for APL, which still requires multicentre validation. Therefore, our results showed the possibility of utilizing lnc-LOC as a noninvasive target for monitoring APL recurrence in patients with clinical suspicion of extramedullary relapse that may not be amenable to biopsy. In addition, many researchers have proposed that various regulatory correlations exist between lncRNAs and miRNAs. According to our previous study, lnc-LOC may change the function of miR-638 to initiate, maintain, and develop APL. Regrettably, the specific mechanism remains to be further studied. However, there are some limitations to the present study, including a relatively small sample size (especially high-risk cases), individual heterogeneity, and shorter observation duration. Therefore, our results still require multicentre validation. Meanwhile, whether PB lnc-LOC can be used to predict clinical outcomes, such as the relapse rates and survival of APL patients, needs to be further studied by expanding the sample size.

In conclusion, this study provides the first evidence that lnc-LOC may be a circulating biomarker for APL, including for diagnosis, treatment response, and MRD surveillance. Our study provided evidence that PB lnc-LOC could serve as a potential biomarker for APL, which set a precedent in the field of lncRNA noninvasive biomarkers of APL.

## Supplementary Information

**Additional file 1: Supplemental Figure 1.** The representative lnc-LOC and GAPDH amplification plots by qRT-PCR based on TaqMan probe. a The amplification plot of lnc-LOC from APL patients. b The amplification plot of GAPDH from APL patients. c The amplification plot of lnc-LOC from non-APL AML patients. d The amplification plot of GAPDH from non-APL AML patients. e The amplification plot of lnc-LOC from healthy donors. f The amplification plot of GAPDH from healthy donors. The x-axis represents the cycle number, and the y-axis represents the relative change in the fluorescence values. The threshold of amplification plot is set at 0.100.

**Additional file 2: Supplemental Figure 2.** Expression of lnc-LOC in non-APL cells treated with ATRA. lnc-LOC expression in all cell lines were measured by qRT–PCR. qRT–PCR results are expressed as mean ± standard deviation.

**Additional file 3: Supplemental Table 1.** Genetic characteristic of lnc-LOC.

## Data Availability

The datasets generated during and analyzed during the current study are not publicly available due to patient privacy reasons but are available from the corresponding author on reasonable request.

## Abbreviations

APL: Acute promyelocytic leukaemia
PML: Promyelocytic
RARα: Retinol receptor alpha
ATRA: All-trans retinoic acid
MRD: Minimal residual disease
BM: Bone marrow
PB: Peripheral blood
AML: Acute myeloid leukaemia
lncRNAs: Long noncoding RNAs
lnc-LOC: Long noncoding RNA LOC100506453
qRT–PCR: Quantitative real-time PCR
CR: Complete remission
DMSO: Dimethyl sulfoxide
ROC: Receiver operating characteristic
CG: Control gene
NCN: Normalized copy number
bp: Base pair
AUCs: Areas under the curves
FISH: Fluorescence in situ hybridization
