# Supplementary figures and images for: Circulating lnc-LOC as a novel noninvasive biomarker in the treatment surveillance of acute promyelocytic leukaemia
Source: BMC Cancer. 2022 May 2;22:481. doi: 10.1186/s12885-022-09621-1 (PMC9059359; doi:10.1186/s12885-022-09621-1)

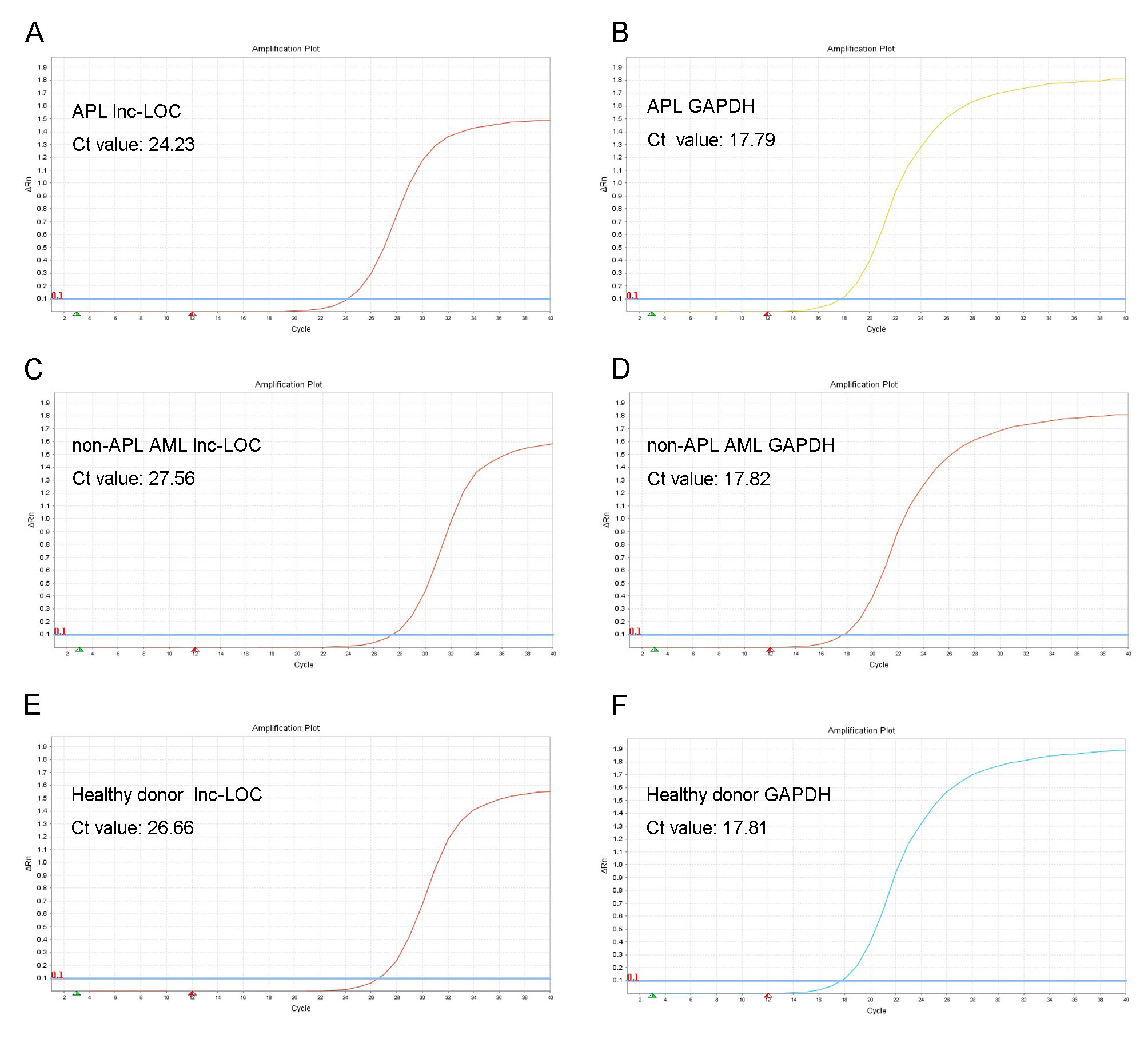

Supplement: Supplementary file 1 — Additional file 1: Supplemental Figure 1. The representative lnc-LOC and GAPDH amplification plots by qRT-PCR based on TaqMan probe. a The amplification plot of lnc-LOC from APL patients. b The amplification plot of GAPDH from APL patients. c The amplification plot of lnc-LOC from non-APL AML patients. d The amplification plot of GAPDH from non-APL AML patients. e The amplification plot of lnc-LOC from healthy donors. f The amplification plot of GAPDH from healthy donors. The x-axis represents the cycle number, and the y-axis represents the relative change in the fluorescence values. The threshold of amplification plot is set at 0.100. [file 12885_2022_9621_MOESM1_ESM.jpg]

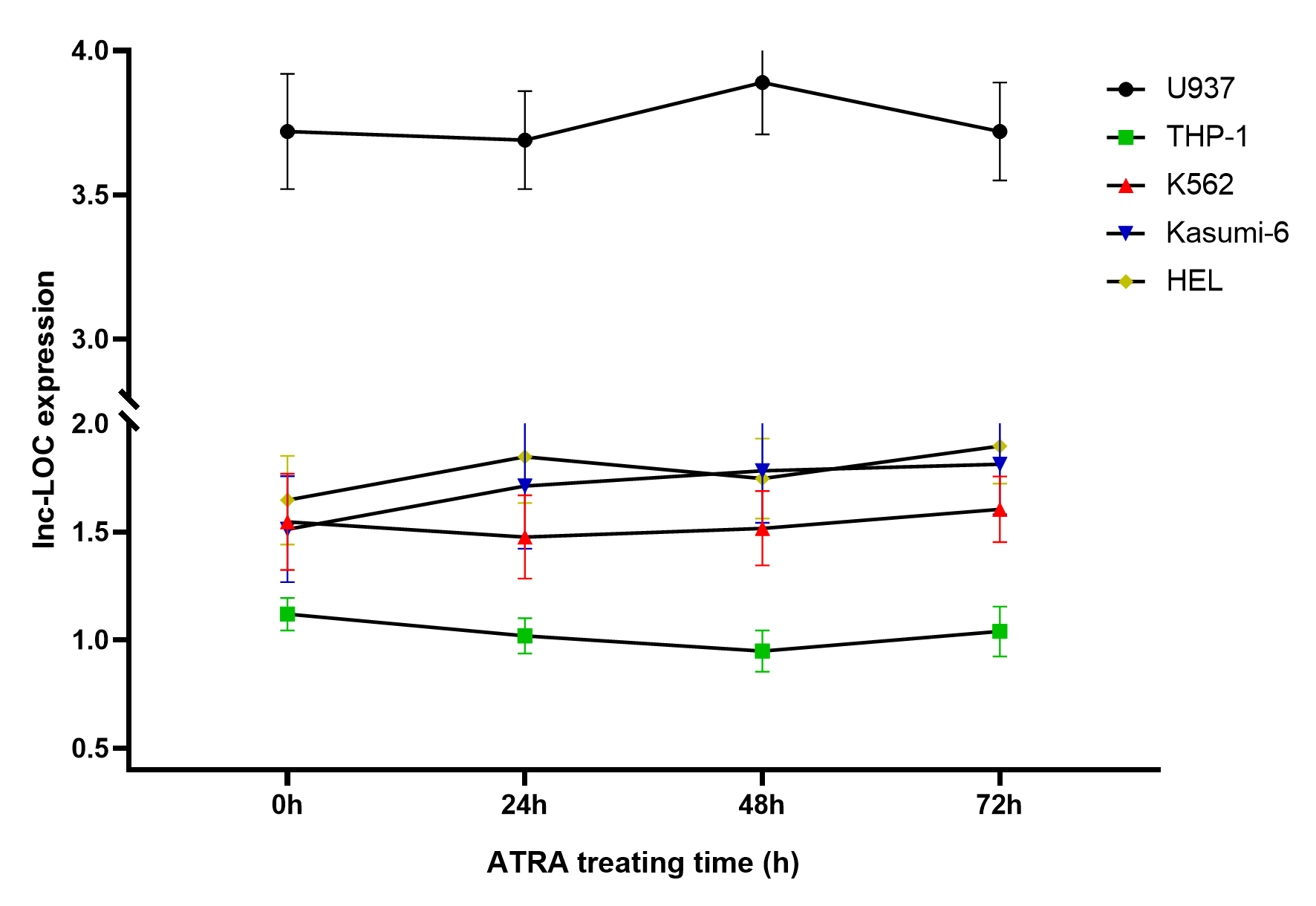

Supplement: Supplementary file 2 — Additional file 2: Supplemental Figure 2. Expression of lnc-LOC in non-APL cells treated with ATRA. lnc-LOC expression in all cell lines were measured by qRT–PCR. qRT–PCR results are expressed as mean ± standard deviation. [file 12885_2022_9621_MOESM2_ESM.tif]
