# Supplementary material for: Circulating lnc-LOC as a novel noninvasive biomarker in the treatment surveillance of acute promyelocytic leukaemia
Source: BMC Cancer. 2022 May 2;22:481. doi: 10.1186/s12885-022-09621-1 (PMC9059359; doi:10.1186/s12885-022-09621-1)
Supplement: Supplementary file 3 — Additional file 3: Supplemental Table 1. Genetic characteristic of lnc-LOC. [file 12885_2022_9621_MOESM3_ESM.docx]

| **Supplemental Table 1** Genetic characteristic of lnc-LOC | |
| --- | --- |
| **Item** | **Genetic characteristics** |
| Gene | A product of gene ENSG00000205663.5 |
| Gene Symbol | LOC100506453 |
| Gene Accession | ENST00000424415 |
| Gene Description | uncharacterized LOC100506453 |
| Location | Chromosome X: 3,809,479-3,820,041 reverse strand |
| Transcript ID | ENST00000424415.1 |
| About this transcript | This transcript has 3 exons, is associated with 286 variant alleles and maps to 584 oligo probes |
| Locus type | NonCoding |
| Base pair | 2194 |
| Protein | No protein |
| Biotype | lincRNA |
| Abbreviations: *lnc-LOC*, long noncoding RNA LOC100506453; *ID,* identification. | |
